# Fascia iliaca compartment block can reduce the incidence of early post-operative cognitive impairment in elderly patients with high-risk hip replacement

**DOI:** 10.3389/fnagi.2022.1025545

**Published:** 2022-12-05

**Authors:** Li Tang, Bo Li, Shun Guo, Xiaoyong Zhao, Binbin He, Weiwei Liu, Rui Xia

**Affiliations:** Department of Anesthesiology, First Affiliated Hospital of Yangtze University, Jingzhou, China

**Keywords:** fascia iliaca compartment block, hypobaric spinal anesthesia, POCD, elderly patients, high-risk hip arthroplasty

## Abstract

**Objective:**

In this study, we aimed to observe the effects of ultrasound-guided fascia iliaca compartment block (FICB) combined with hypobaric spinal anesthesia on post-operative pain and cognitive function in elderly patients with high-risk hip replacement.

**Methods:**

A total of 84 elderly patients—aged 65–85 years, with American Society of Anesthesiologists physical status III–IV, and scheduled for hip arthroplasty between September 2021 and May 2022—were selected. One or more organs with moderate to severe impairment were included in all patients. The patients were randomly divided into a hypobaric spinal anesthesia group (group C, control group) and an ultrasound-guided FICB combined with hypobaric spinal anesthesia group (group E, experimental group). Group C was given 3.5 mL of 0.32% ropivacaine hypobaric spinal anesthesia, and group E received ultrasound-guided FICB combined with 3.5 mL of 0.32% ropivacaine hypobaric spinal anesthesia. The patients were compared using the visual analog scale (VAS) for pain, Harris hip function score, and simple Mini-Mental State Examination (MMSE) scale. Blood β-amyloid (Aβ) and neuronal microtubule-associated protein (tau) levels were measured. We compared intraoperative conditions and post-operative complications between the two groups to assess the effects of FICB combined with hypobaric spinal anesthesia on post-operative pain and cognitive function in elderly patients with high-risk hip replacement.

**Results:**

At 1 and 3 days after the operation, patients in group C had significantly higher VAS and lower MMES scores than those in group E. The differences were statistically significant at 1 (*P* < 0.01) and 3 (*P* < 0.05) days after the operation. At 1 day after operation, the Harris score of patients in group C was significantly lower than that of patients in group E (*P* < 0.05). The Aβ and tau levels of patients in group C were significantly higher than those of patients in group E at 1 day after the operation (*P* < 0.01). The Aβ levels of patients in group C were significantly higher than those of patients in group E at 3 days after the operation (*P* < 0.05). The intraoperative conditions and post-operative complication rates did not differ significantly between the two groups. At 1 day before and 5 days after the operation, there was no difference in any of the indicators.

**Conclusion:**

By lowering pain and managing Aβ and tau protein concentrations, FICB can successfully lower the incidence of early post-operative cognitive impairment in elderly patients with high-risk hip replacement.

**Clinical trial registration:**

www.chictr.org.cn, identifier: ChiCTR2100051162.

## Introduction

In individuals without pre-existing mental illness, post-operative cognitive dysfunction (POCD) is characterized by cerebral dysfunction, including disorientation, anxiety, memory loss, and other aspects of impaired brain function. POCD is a common post-operative complication of the central nervous system, which frequently affects elderly patients. Elderly patients frequently present with several systemic and degenerative diseases, such as hypertension, diabetes mellitus, chronic obstructive pulmonary disease (COPD), coronary artery disease, valvular disease, various cardiac arrhythmias, and cardiac insufficiency, which could be the physiological factors that predispose elderly patients to post-operative cognitive dysfunction (POCD) (Işik, 2015).

POCD mostly occurs in the first 4 days following surgery, and most individuals recover quickly. However, the pathogenesis of POCD is not well-understood. Several factors, such as advanced age, cardiovascular illness, endocrine system disease, history of surgical anesthesia, and neurological alterations, are associated with the development of POCD. Pain may be one of the variables that influence altered cognitive performance and promote its deterioration (Chen et al., 2019; Ding et al., 2021). The occurrence of POCD can be reduced through proper pain evaluation, sufficient analgesia, and prevention of the transition from acute to chronic pain (Huai et al., 2021; Vacas et al., 2021).

Hip replacement is currently a widely used and effective treatment for patients with femoral neck fractures or comminuted acetabular fractures, both of which are frequently accompanied by considerable discomfort and pain. Elderly individuals, especially high-risk elderly patients with underlying cardiac and cerebrovascular disorders, are more likely to develop POCD following hip arthroplasty. According to previous research, deciding on the appropriate anesthetic and proper management of post-operative pain can help lower the risk of POCD. The choice of anesthesia for total hip arthroplasty is related to patient prognosis and desired outcomes (Aksoy et al., 2014; Mei et al., 2017).

Hypobaric unilateral spinal anesthesia has obvious advantages over other anesthetic modalities in hip replacement surgery for elderly patients, such as stable vital signs, less need for drugs, and accurate and rapid analgesic effect. However, the anesthetic procedure requires a special knee–chest position, which results in relatively poor patient compliance and often increases the difficulty of the anesthetic procedure because of pain (Kim and Ahn, 2005; Xu et al., 2017). When performing hip replacement surgery in high-risk older patients, finding a pleasant and satisfactory anesthetic procedure that offers sufficient analgesia without resulting in POCD is crucial.

Fascia iliaca compartment block (FICB) provides optimal analgesia for patients. When local anesthetics are successful in pain reduction during positioning, this considerably increases patient cooperation and satisfaction and lessens the difficulty of spinal anesthesia puncture (Pu et al., 2021). The iliac fascia gap is an external manifestation of the spinal plexus (Kacha et al., 2018; Wennberg et al., 2019). In recent years, with the rapid development of ultrasound visualization technology in the field of anesthesia, peripheral nerve block techniques have become widely used in clinical practice (Okereke and Abdelmonem, 2021).

β-Amyloid (Aβ) and neuronal microtubule-associated protein (tau) are hallmark proteins associated with Alzheimer's disease. Aβ and tau have also been reported as major contributors to cognitive dysfunction (Wu et al., 2021). In patients undergoing hip arthroplasty, general anesthesia is thought to considerably increase Aβ and tau protein concentrations, which may contribute to early development of POCD (Xie et al., 2014; Zhang et al., 2018). Spinal anesthesia can effectively reduce the incidence of early POCD by controlling Aβ and tau protein concentrations; however, the effect of iliac fascia gap block on Aβ and tau protein concentrations and whether it increases the incidence of POCD need further investigation.

## Materials and methods

### Trial design

**Figure 1:**
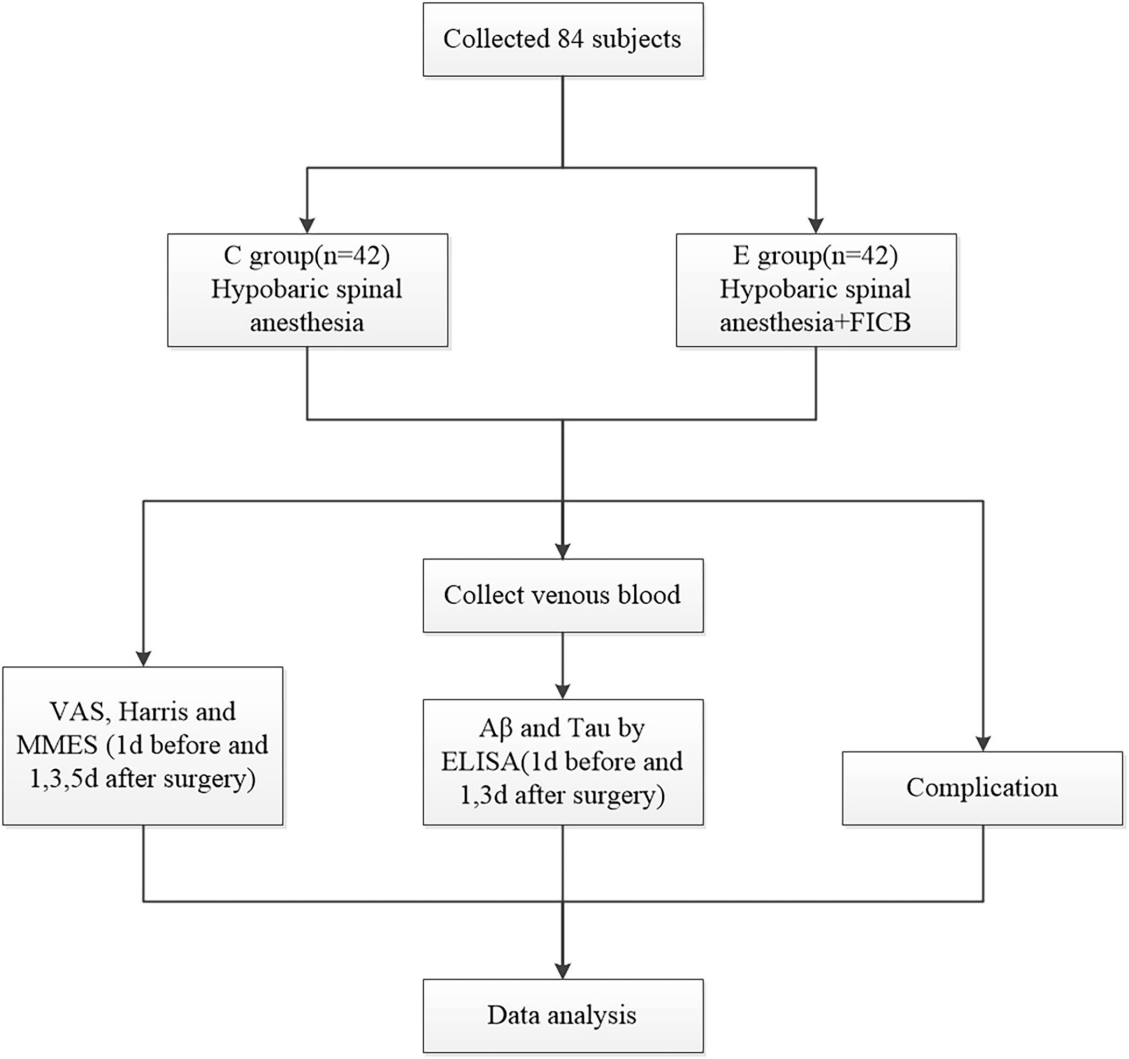
Research flowchart.

A total of 84 cases were randomly divided into a hypobaric spinal anesthesia group (group C, control group) and an ultrasound-guided FICB combined with hypobaric spinal anesthesia group (group E, experimental group). The visual analog scale (VAS) for pain, Harris hip function score, and neuropsychiatric function using the Mini-Mental State Examination (MMSE) scale were assessed on the 1st day prior to surgery and the 1st, 3rd, and 5th days following surgery. Blood Aβ and neuronal microtubule-associated protein (tau) levels were measured using enzyme-linked immunosorbent assay (ELISA). Post-operative complications were observed in both groups. The analgesic strategy was blinded to the researchers who analyzed the scores. The study was approved by the hospital medical ethics committee, and all patients signed an informed consent form before undergoing the anesthesia procedure. All anesthesia procedures and management were performed by the same experienced anesthesiologist. A research flowchart is presented in Figure 1.

### Participants

The inclusion criteria (Rapchuk and Glover, 2013) were as follows: age 65–85 years, ASA III–IV, combined moderate or severe functional impairment of one or more organs, elementary school education or above, and a pre-operative MMSE score ≥24. The exclusion criteria include Alzheimer's disease, absolute contraindication to spinal anesthesia, allergy to local anesthetics, a history of drug abuse or drug addiction, severe audiovisual disorders, more than two successive surgeries, long-term pre-operative analgesics, and intellectual disability or mental illness. The medical and nursing staff communicated with the patients 1 day before the patients underwent hip replacement surgery to ascertain a full understanding of the experimental process and precautions, taught the patients how to evaluate pain using the VAS method, and informed the patients and their escorts regarding the use of an analgesic pump and precautions after the operation.

Prior to the surgery, all patients received medical care, and their physiological functions were optimized. Patients with a history of combined hypertension, coronary artery disease, and pulmonary heart disease, should normally take anti-hypertensive drugs, cor pulmonale medicine, and anti-arrhythmic agents until the morning of surgery. Antiplatelet medications were bridged with low-molecular-weight heparin for anticoagulation 7 days before surgery; however, neither anticholinergics nor sedatives were administered. Low-molecular weight heparin should be discontinued after 12 h, and the coagulation function should be normal. Following admission, routine blood pressure, heart rate, temperature, electrocardiogram, and oxygen saturation measurements were performed using a Philips MP50, and 3 L/min of oxygen was constantly supplied using a mask. A radial artery puncture was used to monitor invasive blood pressure after venous access was opened.

### Interventions

A measure of 3.5 mL of 0.32% ropivacaine was used for hypobaric spinal anesthesia in group C, and 25 mL of 0.3% ropivacaine was used for ultrasound-guided FICB in combination with hypobaric spinal anesthesia in group E (Cantürk et al., 2012). Spinal anesthesia in group E was the same as that in group C.

In group E, FICB was performed after invasive blood pressure was acquired. After the FICB was placed 15 min later, lumbar anesthesia was initiated. In group C, hypobaric spinal lumbar anesthesia was administered.

An ultrasound-guided intraplanar superior inguinal ligament approach was used. The specific methods were as follows (Desmet et al., 2017): An ultrasound probe (Sonosite EDGE) was placed longitudinally on the inguinal ligament, and ultrasound imaging of the anterior superior iliac spine was performed in the sagittal plane. The ultrasound probe slowly slid medially until the fascia iliac, sartorius, iliopsoas, and internal oblique muscles were obtained in the same image. Ultrasound imaging was used to distinguish between the internal abdominal oblique, sartorius, and anterior superior iliac spine. The puncture needle was placed at a 45° angle with the skin, and the needle was inserted from the tail end of the ultrasound probe using the in-plane injection method. Under real-time ultrasound monitoring, the needle tip (German Beltran, Stimuplex D) reached the fascia iliac space, and no blood was found by backpumping. After injection of 2–3 mL of normal saline, the liquid diffused into the fascia iliac space, indicating that the position of the needle tip was correct. Subsequently, 25 mL of 0.3% ropivacaine was administered over the course of 3 min after the syringe was aspirated to ensure that the needle did not pierce any arteries.

Hypobaric spinal anesthesia method: When using the hypobaric spinal anesthetic technique, the patient should be asked to hold the knee with both hands, with the thighs as close to the abdomen as possible and the head bent to the chest as closely as possible. The cerebrospinal fluid flow was observed to be unhindered following intravertebral puncture, and 0.32% ropivacaine 3.5 mL (0.75% ropivacaine 1.5 mL + sterile injection water 2 mL) was administered at a rate of 0.2 mL/s. After administration, the affected side was kept upward, and the level of anesthesia was adjusted to ~T10 for 15 min. The vital signs of the patients were closely monitored. If the blood pressure of the patients was below 30% of the basal blood pressure, 5–10 mg ephedrine hydrochloride was injected. If the patients had bradycardia during the operation and the heart rate was below 50 beats/min, atropine 0.25–0.5 mg was administered.

At the end of the operation, a PCIA device was installed in both groups. For PCIA, the control group had the same parameters as that of the experimental group. Both groups were treated using an intravenous analgesic pump, 2 μg/kg of sufentanil (Yichang Humanwell Pharmaceutical), 5 mg of tropisetron (Jiangsu Hengrui Medicine), and normal saline diluted to 100 mL. Treatment parameters were set as follows: loading dose: 2 mL; continuous infusion volume: 2 mL/h; automatic control volume: 1.5 mL/ time; and lock each press interval: 15 min. Patients in both groups were administered an intravenous infusion of 100 mg of flurbiprofen axetil on the 1st and the 2nd day after the operation.

### Sample collection and evaluation

#### Harris hip score criteria

The hip grade and efficacy score were interpreted as follows: A score of 91–100 with a pain score of 40 was considered excellent; a score of 76–90 with a pain score of ≥ 30 was good; a score of 50–75 with a pain score of ≥20 was fair; and a score of ≤ 49 with a pain score ≤ 10 was poor (Xiao et al., 2020).

#### MMSE scoring criteria

Patients with high scores were better than those with low scores, and this experiment only compared the differences in total scores. If the primary school culture (≤6 years of education) group scored ≤20 points and middle school and above (≥6 years of education) group scored ≤24 points, cognitive function deficits were determined (Postler et al., 2011).

#### Enzyme-linked immunosorbent assay (ELISA) for Aβ and tau protein levels

Peripheral blood (4 mL) was drawn from the patients in ethylenediamine tetraacetic acid anticoagulation tubes at 1 day before, 1 and 3 days after, and in the early morning of the surgery (Uzoigwe et al., 2020). The blood sample was centrifuged for 10 min (2,000 rpm), and the plasma was collected for the detection of blood Aβ and tau levels by double-antibody sandwich ELISA.

### Statistical analysis

The findings were analyzed by using SPSS software (version 21.0), and descriptive statistics were presented as means and standard deviations, while non-normally distributed measures were presented as medians and quartiles. A variance test was used to examine normally distributed group data, and a non-parametric test was used to examine non-normally distributed data. A paired-sample *t*-test was used to compare patients' conditions before and after treatment. The chi-square test was used to compare the classified categories. The significance level of the difference was set at α = 0.05. Differences were considered statistically significant at a *p*-value of <0.05.

## Results

### Comparison of the general conditions before treatment

A total of 84 patients were included in this study. Of these, 42 patients were assigned to group C (17 men and 25 women), and 42 patients were assigned to group E (18 men and 24 women). Table 1 shows a general comparison of the patients in the two groups. The findings indicated that sex, age, and educational attainment did not differ significantly between the two groups, making them equivalent. Moreover, there were no significant differences in comorbidities between the two groups (Table 2).

**Table 1:** Comparison of general conditions of the two groups.

**Gender: number (%)**	**Group C (*n* = 42)**	**Group E (*n* = 42)**	**Aggregate**	**Statistic**	***p*-value**
Male	17 (40.47)	18 (42.86)	35 (41.67)	χ^2^ = 0.049	0.825
Female	25 (59.23)	24 (57.14)	49 (58.33)		
Age ($\bar{x}\pm s$)	78.53 ± 8.66	77.62 ± 7.61		*t* = 0.512	0.612

**Table 2:** Comparison of comorbidities of the two groups.

**Number (%)**	**Hypertension**	**Diabetes**	**Coronary heart disease**	**Arrhythmia**	**COPD**
Group C	25 (59.52)	9 (21.43)	5 (11.90)	6 (14.26)	12 (28.57)
Group E	24 (57.13)	8 (19.05)	8 (19.05)	10 (23.81)	5 (11.90)
χ^2^	0.049	0.074	0.812	1.469	3.64
*P*	0.825	0.786	0.368	0.226	0.06

### Comparison of VAS, Harris, and MMES scores between the two groups

At 1 and 3 days after the operation, patients in group C had significantly higher VAS scores and lower MMES scores than those in group E. The differences were statistically significant at 1 (*P* < 0.01) and 3 days (*P* < 0.05) after the operation. At 1 day after the operation, the Harris score of the patients in group C was significantly lower than that in group E (*P* < 0.05). At 1 day before and 5 days after the surgery, there were no significant differences in the VAS, Harris, and MMES scores between the two groups (*P* > 0.05) (Tables 3–5).

**Table 3:** Comparison of VAS of the two groups.

	**Group C (*n* = 42)**	**Group E (*n* = 42)**	***t***	***p*-value**
1 d before surgery	5.45 ± 0.87	5.42 ± 0.90	0.155	0.877
1 d after surgery	3.48 ± 0.75	3.03 ± 0.59**	3.056	0.004
3 d after surgery	2.95 ± 0.51	2.67 ± 0.49*	2.566	0.014
5 d after surgery	1.60 ± 0.22	1.55 ± 0.21	1.065	0.293

Compared with group C,

* P < 0.05,

** P < 0.01.

**Table 4:** Comparison of Harris of the two groups.

	**Group C (*n* = 42)**	**Group E (*n* = 42)**	***t***	***p*-value**
1 d before surgery	14.06 ± 2.09	14.00 ± 2.16	0.129	0.900
1 d after surgery	40.44 ± 3.92	43.51 ± 5.67*	−2.679	0.011
3 d after surgery	43.25 ± 5.26	45.29 ± 5.30	−1.771	0.084
5 d after surgery	65.60 ± 4.67	66.10 ± 4.71	−0.489	0.628

Compared with group C,

* P < 0.05.

**Table 5:** Comparison of the score of MMSE.

	**Group C (*n* = 42)**	**Group E (*n* = 42)**	***t***	***p*-value**
1 d before surgery	22.61 ± 4.63	22.52 ± 4.55	0.090	0.929
1 d after surgery	22.51 ± 4.60	25.20 ± 4.10**	−2.830	0.006
3 d after surgery	23.14 ± 3.61	24.89 ± 4.02*	−2.100	0.039
5 d after surgery	24.30 ± 2.34	24.57 ± 1.94	−0.576	0.566

Compared with group C,

* P < 0.05,

** P < 0.01.

### Comparison of Aβ and tau in the two groups

The Aβ and tau levels of patients in group C were significantly higher than those of patients in group E 1 day after the operation (*P* < 0.01). The Aβ levels of patients in group C were significantly higher than those in group E at 3 days after the operation (*P* < 0.05) (Table 6).

**Table 6:** Comparison of the expression levels of Aβ and tau.

		**Group C (*n* = 42)**	**Group E (*n* = 42)**	***t***	***p*-value**
1 d before surgery	Aβ (μg/L)	484.8 ± 50.5	480.0 ± 48.7	0.443	0.66
	Tau (ng/L)	70.1 ± 7.6	68.3 ± 7.5	1.093	0.281
1 d after surgery	Aβ (μg/L)	892.4 ± 100.1	678.7 ± 83.3**	10.63	0.005
	Tau (ng/L)	98.1 ± 10.1	90.2 ± 9.4**	3.711	0.001
3 d after surgery	Aβ (μg/L)	526.7 ± 40.5	500.6 ± 50.4*	2.616	0.012
	Tau (ng/L)	80.8 ± 15.6	74.6 ± 13.5	1.948	0.058

Compared with group C,

* P < 0.05,

** P < 0.01.

### Comparison of intraoperative conditions

There were no significant differences in operation time, blood loss, infusion volume, and urine volume between the two groups. The anesthesia duration in group E was longer than that in group C (*P* < 0.05) (Table 7).

**Table 7:** Comparison of intraoperative conditions of the two groups.

	**Group C (*n* = 42)**	**Group E (*n* = 42)**	***t***	***p*-value**
Operation time (min)	70.5 ± 25.7	72.6 ± 23.0	−0.395	0.694
Blood loss (mL)	342.9 ± 78.2	323.5 ± 86.8	1.077	0.285
Infusion volume (mL)	900.7 ± 250.0	1000.3 ± 284.6	−1.704	0.092
Urine volume (mL)	250.3 ± 61.1	261.4 ± 74.9	−0.744	0.459
Anesthesia time (min)	100.8 ± 36.3	120.4 ± 45.7*	−2.177	0.032

Compared with group C,

* P < 0.05.

### Comparison of the rates of complication

There were four cases of post-operative nausea and vomiting (PONV), eight cases of chills, and two cases of hypotension in patients in group C. There were six cases of nausea and vomiting, six cases of chills, and three cases of hypotension in group E. No statistical difference existed (Table 8).

**Table 8:** Comparison of complication rates.

**Number (%)**	**PONV**	**Chill**	**Hypotension**
Group C	4 (9.52)	8 (19.05)	2 (4.76)
Group E	6 (14.26)	6 (14.26)	3 (7.14)
χ^2^	0.454	0.343	0.213
*P*	0.50	0.558	0.644

## Discussion

In older patients, following hip arthroplasty, POCD frequently clinically presents as memory loss, disorientation, psychomotor derangement, and depression (Shi et al., 2015; Ertürk et al., 2021). POCD also makes it harder for patients to recover, increases their financial burden and mental stress, and worsens their suffering. Without prompt care, dementia may develop in severe cases and put patients' lives in danger (Tzimas et al., 2018; Ehsani et al., 2020).

General anesthesia is more difficult than spinal anesthesia, and the administration of anesthetics and vasoactive medications has a significant impact on all systems (Lin et al., 2020). Significant rates of pulmonary problems and pain following surgery result in high costs and prolonged hospital stay (Uzoigwe et al., 2020). Patients under general anesthesia are more likely than those under spinal anesthesia to experience early POCD (Mason et al., 2010; Yeung et al., 2016). Owing to the aforementioned reasons, the clinical use of general anesthesia in patients undergoing hip surgery is uncommon.

Hypobaric anesthesia provides a more rapid onset of action than isobaric and hyperbaric spinal anesthesia, as well as easy plane adjustment and control, minimal impact on the patient's hemodynamics, and a precise anesthetic effect, which can enhance post-operative recovery (Vergari et al., 2017). Patients with hip fractures who have lumbar anesthesia experience frequent intense pain at the fracture site, which results in a number of pathophysiological changes, such as elevated blood pressure, elevated heart rate, and significant release of stress hormones. For simplicity, safety, and efficiency, the iliac fascia gap can successfully reduce discomfort caused by postural alterations and post-operative analgesia (Kearns et al., 2011).

### Effect of nerve block on pain scores and hip function scores

Multiple factors including joint function, post-operative mood, sleep, and stress affect post-operative pain (Krych et al., 2014; Verbeek et al., 2021). The VAS scores of patients in group C were significantly higher than those of patients in group E on the 1st (*p* < 0.01) and the 3rd day after the surgery (*p* < 0.05), indicating a prolonged duration of action of ultrasound-guided FICB with 25 mL of 0.3% ropivacaine. Within 24 h of surgery, there was a noticeable effect, which may have tapered off 24 h later. Therefore, ultrasound visualization-guided FICB is beneficial for early post-operative pain during hip surgery (Fujihara et al., 2013).

The Harris hip function score, a more reliable hip function score scale, divides hip function into four categories using numerical quantification (Høgh et al., 2008). Patients in group C had substantially higher Harris scores than those in group E on the 1st post-operative day (*P* < 0.05). The superior analgesia in group E compared with group C could be responsible for the higher Harris function score in group E than in group C on post-operative day 1. Bed exercises and functional recovery were facilitated in patients with mild pain.

### Effect of nerve block on MMSE, Aβ, and tau content

The MMSE is a straightforward test scale used to evaluate cognitive impairment and measures cognitive abilities such as orientation, memory, language, math, and attention. It can fully, correctly, and quickly reflect an individual's mental health and level of cognitive impairment.

The MMSE used in this study can assess the patients' cognitive function in the early post-operative period and is more authoritative. In the results, the MMSE scores of patients in group C were significantly lower than those of patients in group E on the 1st day after surgery, and the difference was statistically significant (*P* < 0.05). We concluded that the overall cognitive function of group E was better than that of group C according to those with higher scores than those with lower scores.

Aβ protein has a molecular weight of ~4 kDa and is derived from the hydrolysis of the β-amyloid precursor protein (Li et al., 2014; Tomaszewski, 2015). According to previous reports, after precipitating and building up in the cell matrix, Aβ has a severe neurotoxic effect, which increases the risk of neuronal death and neurodegeneration (Wu et al., 2018). The cholinergic system is closely tied to human learning, memory, and cognitive function, and elevated Aβ is detrimental to the cholinergic system; therefore, elevated Aβ is a substantial contributor to cognitive dysfunction (Lin et al., 2020).

Tau is the most abundant microtubule-associated protein and mainly binds to microtubule proteins to promote their polymerization to form microtubules, which are necessary to maintain the transport of substances between the neuronal cell cytosol and axons (Shen et al., 2016; Liang et al., 2022). In this study, the levels of Aβ and tau were significantly higher in group C than in group E on post-operative day 1 (*P* < 0.01). The Aβ levels of the patients in group C were significantly higher than those in group E on post-operative day 3 (*P* < 0.05). This indicates that ultrasound-guided iliac fascia gap block may be beneficial in improving cognitive function scores and reducing the occurrence of post-operative cognitive function in elderly patients undergoing hip surgery, mainly by reducing post-operative pain and controlling the levels of Aβ and tau (Perrier et al., 2010; Ji et al., 2013).

## Conclusion

In conclusion, increased Aβ and tau protein concentrations may contribute to the development of cognitive dysfunction. By lowering pain and managing Aβ and tau protein concentrations, FICB can successfully lower the incidence of early post-operative cognitive impairment in elderly patients with high-risk hip replacement.

## Data availability statement

The original contributions presented in the study are included in the article/supplementary material, further inquiries can be directed to the corresponding author/s.

## Ethics statement

The studies involving human participants were reviewed and approved by the First Peoples' Hospital of Jingzhou Medical Ethics Committee. The patients/participants provided their written informed consent to participate in this study.

## Author contributions

LT, RX, and BL participated in the design of the study. XZ and BH carried out the experiment and analyzed the data. BL and WL collected the sample. RX and XZ evaluated the sample of the subjects. LT and WL wrote the manuscript. All authors contributed to the article and approved the submitted version.

## Funding

The present work was supported by grants from the China National Key Research and Development (Program No. 2020YFC2009002).

## Conflict of interest

The authors declare that the research was conducted in the absence of any commercial or financial relationships that could be construed as a potential conflict of interest.

## Publisher's note

All claims expressed in this article are solely those of the authors and do not necessarily represent those of their affiliated organizations, or those of the publisher, the editors and the reviewers. Any product that may be evaluated in this article, or claim that may be made by its manufacturer, is not guaranteed or endorsed by the publisher.
